# A multilevel developmental psychopathology model of childbirth and the perinatal transition

**DOI:** 10.1017/S0954579422001389

**Published:** 2023-01-26

**Authors:** Parisa R. Kaliush, Elisabeth Conradt, Patricia K. Kerig, Paula G. Williams, Sheila E. Crowell

**Affiliations:** 1 Department of Psychology, University of Utah, 380 South 1530 East, BEH S 502, Salt Lake City, UT 84112, USA,; 2 Department of Psychiatry and Behavioral Sciences, Duke University, Durham, NC 27701, USA,; 3 Department of Psychiatry, University of Utah, Salt Lake City, UT 84108, USA; 4 Department of Obstetrics and Gynecology, University of Utah, Salt Lake City, UT 84132, USA

**Keywords:** Childbirth, conceptual model, developmental psychopathology, perinatal period, traumatic childbirth

## Abstract

Despite recent applications of a developmental psychopathology perspective to the perinatal period, these conceptualizations have largely ignored the role that childbirth plays in the perinatal transition. Thus, we present a conceptual model of childbirth as a bridge between prenatal and postnatal health. We argue that biopsychosocial factors during pregnancy influence postnatal health trajectories both directly and indirectly through childbirth experiences, and we focus our review on those indirect effects. In order to frame our model within a developmental psychopathology lens, we first describe “typical” biopsychosocial aspects of pregnancy and childbirth. Then, we explore ways in which these processes may deviate from the norm to result in adverse or traumatic childbirth experiences. We briefly describe early postnatal health trajectories that may follow from these birth experiences, including those which are adaptive despite traumatic childbirth, and we conclude with implications for research and clinical practice. We intend for our model to illuminate the importance of including childbirth in multilevel perinatal research. This advancement is critical for reducing perinatal health disparities and promoting health and well-being among birthing parents and their children.

Within the last decade, the perinatal period has rapidly become a high-priority area of research (Howard & Khalifeh, 2020). There is increasing awareness of vast and persistent maternal health disparities in the United States, as well as intergenerational consequences and economic costs of inadequate perinatal healthcare (Glazer & Howell, 2021). The transition from pregnancy to postpartum often is described as one of the most complicated, meaningful, and challenging developmental transitions in the human life span. Thus, it is ideal timing to apply a developmental psychopathology perspective to this life phase because this framework emphasizes longitudinal, multilevel investigations of both adaptive and maladaptive health (Cicchetti & Rogosch, 1996; Cicchetti & Toth, 2009). Indeed, there have been recent applications of this framework to the perinatal period (e.g., Davis & Narayan, 2020; Doyle & Cicchetti, 2018; Glynn et al., 2018; Goodman & Dimidjian, 2012), which hold promise for advancing research and healthcare practices during this critical life phase.

In spite of these conceptual and empirical advancements in perinatal research, childbirth – a critical component of the perinatal transition – has received limited attention in the developmental psychopathology literature (for a biopsychosocial perspective on pain and social support during childbirth, see Saxbe, 2017). Although childbirth is only a fragment of the entire perinatal period, it is a momentous inflection point with the potential to drastically alter birthing parents’ postnatal health trajectories.^1^ Typical childbirth is characterized by numerous biopsychosocial changes that influence birthing parents’ health, parenting behaviors, and interpersonal relationships. In the context of adverse or traumatic childbirth experiences, the consequences may become even more pervasive and enduring. Thus, childbirth cannot be neglected in developmental psychopathology research on the perinatal period.

In this paper, we present a multilevel developmental psychopathology model of childbirth and the perinatal transition (i.e., pregnancy to early postpartum). Our model is informed by pivotal research on neurobiological changes during the perinatal period (e.g., Davis & Narayan, 2020; Glynn & Sandman, 2011), sociocultural influences on the birth experience (e.g., Cooper Owens & Fett, 2019; Greenwood et al., 2020), and the causes and consequences of childbirth trauma (e.g., Beck et al., 2013). Separately, these literatures have illuminated the sensitive nature of the perinatal period, the transgenerational consequences of implicit and systemic racism, and the prevalence of childbirth trauma. We integrate these literatures within a developmental psychopathology framework in order to deepen scholars’ conceptualizations of childbirth as a pathway by which prenatal experiences may indirectly influence postnatal health outcomes.

A central tenet of developmental psychopathology is that development is dynamic across the life span – individuals fluctuate in continuous and discontinuous ways between adaptive and maladaptive functioning (Cicchetti, 2016). However, even during periods of maladaptive functioning, adaptive coping may be present. Thus, it is critical to study both typical and atypical developmental trajectories, as they are mutually informative. In line with this framework, we first outline typical biopsychosocial aspects of pregnancy and childbirth. Then, we explore ways in which these processes might deviate from normative development – in other words, when biological, psychological, and sociocultural processes interact to result in adverse or traumatic childbirth experiences. We acknowledge that many psychological conceptualizations of “typical” pregnancy and childbirth often reflect the experiences of White, well-educated, financially privileged samples. However, there is a burgeoning literature outside of psychology (e.g., in the fields of midwifery, nursing, public health, sociology) on perinatal and childbirth experiences among minoritized and marginalized populations. We integrate this literature throughout our review, consistent with multicultural perspectives and a developmental psychopathology framework for addressing perinatal health disparities (Causadias, 2013; Conradt et al., 2020).

We illustrate these dynamic perinatal processes in a conceptual model (Figure 1). Our model highlights potential ways in which biological, psychological, and sociocultural factors interact during pregnancy and influence multilevel processes postpartum, both directly and indirectly, through effects on childbirth experiences. This model serves as an illustration of the biopsychosocial processes reviewed in this paper. We conclude with a brief description of early postnatal health trajectories, including those which are adaptive following childbirth trauma, as well as implications for research and healthcare practice.

## “Typical” biopsychosocial processes during pregnancy and childbirth

### Neurobiology and sleep

Birthing parents undergo profound neurobiological and anatomical changes that are necessary for pregnancy, childbirth, and preparation for parenthood (for reviews on these changes, see Glynn & Sandman, 2011; Schaffir, 2016). In fact, hormone exposures during the perinatal period are unmatched compared to any other time in the female life span. Human chorionic gonadotropin levels rise rapidly during pregnancy and facilitate the production of progesterone. As a smooth muscle relaxant, progesterone helps to prevent premature birth, reduce hypertension risk, increase oxygen intake, and promote greater nutrient and energy yields (Edwards et al., 2017; Schaffir, 2016). Also, the conversion of progesterone to allopregnanolone (ALLO) – a neurosteroid implicated in physiologic homeostasis following stress – promotes dampened stress responsivity during late gestation, which protects the birthing parent and fetus from adverse effects of stress exposure (de Weerth & Buitelaar, 2005; Glynn et al., 2001; Schiller et al., 2014). These and other prenatal hormonal changes induce alterations in lipid metabolism and shift gut and vaginal microbial diversity to increase fat storage, prepare birthing parents for lactation, and promote bacterial growth that is associated with positive birth outcomes (Edwards et al., 2017; Prince et al., 2015). Placental corticotropin-releasing hormone triggers cortisol production during pregnancy, which supports fetal maturation and potentiates the onset of labor (Gangestad et al., 2012; Howland et al., 2017). Then, during labor, endogenous opioids regulate oxytocin (a neuropeptide that promotes uterine contractions) and help to blunt pain (Saxbe, 2017).

Prenatal sleep is intimately intertwined with these hormone changes (for a review, see Bazalakova et al., 2014). Due to the soporific effects of rapidly increased progesterone levels, the 1^st^ trimester of pregnancy often is characterized by longer sleep duration. However, with advancing gestational age, rising progesterone and cortisol levels contribute to declines in total overnight and REM sleep, more frequent and longer nighttime awakenings, and increased urinary frequency and heartburn. Oftentimes, sleep patterns during the 3rd trimester reflect clinically significant insomnia, which is endorsed by more than 50% of birthing parents (Facco et al., 2010). Increased daytime napping may preserve total sleep time per 24 hr; however, it is unclear whether daytime sleep fully offsets the neurologic and metabolic consequences of poor nighttime sleep and overall poor sleep quality (Bazalakova et al., 2014). In addition to disruption via hormonal changes, prenatal sleep is disrupted by experiences with physical discomfort and back pain, leg cramps and restlessness, shortness of breath, and fetal movements (Bazalakova et al., 2014; Schaffir, 2016).

### Sociohistorical processes

Perceptions and practices surrounding childbirth and the perinatal transition have shifted over time with evolving societal beliefs and scientific developments. The rise of industrialization and modern medicine during the early 20th century instigated a transition in prenatal care and childbirth from the home – in the presence of female kin and midwives – to the hospital with predominantly White, male doctors (Saxbe, 2017). This transition was accompanied by an increase in surgical births (e.g., Cesarean birth, use of forceps during vaginal birth) and use of anesthesia during labor (Declerq et al., 2013; Hamilton et al., 2015), although this latter phenomenon also has ebbed and flowed with shifting feminist perspectives, such as from championing non-medicalized births in the 1960s to supporting women’s rights to choose “pain-free” births in the 1990s (Skowronski, 2015). Similarly, there have been shifts in the involvement of fathers during prenatal care and childbirth. Whereas, historically, fathers were excluded from pregnancy and childbirth matters, advocacy efforts in the 1960s prompted changes in these standards in the United States, and today, father presence at birth is highly valued (Longworth et al., 2015; Saxbe, 2017).

Western industrialization and scientific progress have been associated with advancements in perinatal healthcare and increased capacity to disseminate perinatal science (Lennon, 2016). However, these advancements have also incited increased perceptions of childbirth as a medically dangerous event (Bondas & Eriksson, 2001; Hunter, 2006; Lennon, 2016). Resultantly, there have been shifts in birthing parents’ experiences with pregnancy and childbirth, such as increased anxiety about adhering to medical recommendations and reduced agency in decision-making. These experiences have become increasingly normalized, and yet are often associated with perceptions of childbirth as traumatic.

## Potentially traumatic childbirth

Expanding from this baseline, albeit non-exhaustive, understanding of biopsychosocial aspects of “typical” pregnancy and childbirth, we next examine what happens when these processes deviate from normative development. In other words, we describe the ways in which “atypical” developmental processes may result in adverse or traumatic childbirth experiences, which then impel multifinal postnatal health trajectories. It is estimated that 9–44% of birthing parents describe their childbirth as traumatic, and a wide range of childbirth experiences have fallen under this description (Alcorn et al., 2010; de Graaff et al., 2018; Grekin & O’Hara, 2014; Yildiz et al., 2017). These experiences include (but are not limited to) emergency surgical births, preterm birth, prolonged and painful childbirth, admission of the infant into the Neonatal Intensive Care Unit (NICU), and “degrading” healthcare experiences, such as medical staff making healthcare decisions without input or consent of the birthing parent (Beck, 2004, p. 32). These broad examples highlight that “traumatic childbirth … lies in the eye of the beholder” (Beck, 2020, pp. 132). What may be perceived by obstetrical healthcare providers as routine, nonlife-threatening delivery may be experienced as traumatic and life-altering by birthing parents. Specific rates of subjective traumatic experiences vary based on samples (e.g., community vs. high-risk), methodology (e.g., self-report vs. diagnostic interviews), and time period (e.g., early vs. later postpartum; Williams et al., 2022; Yildiz et al., 2017), but their prevalence underscores the importance of including childbirth in developmental psychopathology conceptualizations of the perinatal transition.

### Atypical neurobiology, stress responsivity, and sleep

Birthing parents who do not exhibit normative biological changes during pregnancy – namely, increasing hormone levels and dampened stress responsivity during the 3rd trimester – may be at heightened risk for adverse or traumatic childbirth experiences (Dickens et al., 2020). For instance, research has demonstrated that increases in perceived stress across gestation are associated with elevated risk for preterm birth even after controlling for obstetric risk, pregnancy-specific anxiety, and prenatal life events (Glynn et al., 2008). In addition, more pronounced cortisol awakening responsivity during the 3rd trimester has been associated with more subjectively negative childbirth experiences measured within the first few postpartum hours (Alder et al., 2011). Given the role that ALLO plays in restoring homeostasis and dampening stress responsivity, it is likely that atypically low levels of this neurosteroid facilitate the relation between prenatal stress and adverse childbirth outcomes. Excessively low levels of ALLO during 2nd trimester have been associated with postpartum depression (PPD) and anxiety (Osborne et al., 2019; Osborne et al., 2017); however, direct relations between prenatal ALLO levels and childbirth outcomes have not been studied.

Also important to consider are individual differences in prenatal stress responsivity, particularly given that these may be heightened by experiences of racism and thus provide a mechanism to explain racial disparities in childbirth experiences and outcomes (Chaney et al., 2019; Conradt et al., 2020; Glynn & Sandman, 2011; Kramer & Hogue, 2009). Black birthing parents in the United States are two times more likely than White birthing parents to give birth preterm, and they are three times more likely to give birth very preterm (Kramer & Hogue, 2009). Exposure to racism, ranging from interpersonal to structural, is experienced in the body like chronic stress in that it increases sympathetic nervous system activation, upregulates the HPA axis, and results in physiological wear-and-tear (for reviews on this topic, see Chaney et al., 2019; Goosby et al., 2018). Indeed, research has shown that Black birthing parents who endorsed multiple experiences of direct and indirect childhood racism and exhibited excessive increases in diastolic blood pressure over the course of pregnancy were at heightened risk for delivering low birthweight infants (Hilmert et al., 2014).

An emerging body of research has begun exploring atypical vaginal and gut microbial communities during pregnancy as risk factors for traumatic childbirth experiences (Corwin et al., 2017). For instance, Black and Latinx birthing parents are less likely than are White birthing parents to exhibit *Lactobacillus*- dominated vaginal microbial profiles, which may partially explain higher rates of preterm birth among birthing parents of color (Fettweis et al., 2014; Hickey et al., 2012; Hyman et al., 2014; Prince et al., 2015). However, one recent longitudinal study found that increased risk for preterm birth was associated with a sudden decrease in vaginal richness and diversity between the 1st and 2nd trimesters, followed by continued microbial instability throughout gestation (Stout et al., 2017). Thus, risk for potentially traumatic childbirth may be related to sudden, early decreases and subsequent instability in vaginal microbiome diversity, which may occur more commonly among birthing parents who endure chronic stress and/or acute stress earlier in gestation. Similarly, atypical alterations in gut microbial composition over the course of gestation – specifically, premature shifts from anti- to pro-inflammatory states, which initiate labor – have been associated with preterm birth (for a review, see Bayar et al., 2020).

Finally, atypical patterns in prenatal sleep may confer risk for adverse and traumatic childbirth experiences. Severe insomnia and sleep apnea during pregnancy have been associated with prolonged labor, emergency surgical births, and preterm birth (Felder et al., 2017; Paavonen et al., 2017). Excessive sleep deprivation has been associated with high levels of pro-inflammatory serum cytokines among pregnant adults, which may explain the links between poor sleep and adverse childbirth outcomes (Okun et al., 2007; for a review, see Chang et al., 2010). In addition, birthing parents who are especially sleep deprived have reported more pain and discomfort during labor (Chang et al., 2010). Sleep deprivation has been associated with increased pain sensitivity among non-pregnant adults (Schrimpf et al., 2015; Smith et al., 2005), and the association between sleep deprivation and elevated stress hormones may explain this association as it pertains to childbirth pain (Alehagen et al., 2001; Besedovsky et al., 2012).

### Psychosocial risk factors

Psychological and sociocultural factors interact dynamically with birthing parents’ neurobiology and sleep to confer risk for traumatic childbirth experiences. For instance, when conceptualizing atypical stress responsivity as a biological risk, it is pertinent to recognize that birthing parents with histories of chronic life stress are likely to continue experiencing stress during pregnancy, which may compromise their bodies’ abilities to downregulate stress responsivity (Dickens et al., 2020). Unstable housing and food scarcity are two chronic stressors that have been associated with labor complications and preterm birth (Buultjens et al., 2013; Clark et al., 2019; Pantell et al., 2019). Moreover, birthing parents facing food scarcity are more likely to have limited education on and access to healthy prenatal diets, which increases their risk for unhealthy weight during pregnancy. High BMI and obesity during pregnancy are associated with elevated risks for insomnia and sleep apnea, and thus preterm and emergency Cesarean birth (Cnattingius et al., 2013; Dempsey et al., 2005; Loy et al., 2013; Swanson et al., 2020).

Daily experiences with racism and discrimination are additional chronic life stressors that have been associated with atypical prenatal sleep, including poorer sleep quality, more difficulties falling asleep, and more frequent nightmares (Feinstein et al., 2020;Kalmbach et al., 2019). It has been theorized that the links between racism and sleep health may partially explain racial and ethnic disparities in adverse birth experiences, but more research on this topic is needed (Feinstein et al., 2020). One recent study found that pre-pregnancy sleep disturbances among non-Hispanic Black birthing parents predicted fetal growth restriction and preterm birth above and beyond prenatal sleep disturbances (White et al., 2022). This finding highlights the need for developmental conceptualizations and longitudinal research on sleep health and childbirth.

Given bidirectional associations between poor sleep and difficulties with emotion regulation (Littlewood et al., 2017), it is likely that these factors interact to increase risk for traumatic childbirth experiences. Indeed, prenatal sleep deprivation has been associated with elevated fears of labor pain (Hall et al., 2009). Moreover, birthing parents’ experiences with depression and anxiety (both generalized and health-specific) have been found to exacerbate these fears, increase the likelihood of requesting Cesarean birth, and predict prolonged labor above and beyond prenatal substance use and health complications (Dencker et al., 2019; Nilsson et al., 2018; Orr et al., 2002; Polachek et al., 2016; Türkmen et al., 2020). Some research indicates that these psychological factors are more potent predictors of traumatic childbirth than the mode of birth itself (King et al., 2017). Heightened psychological distress increases stress hormones, such as cortisol and epinephrine, that may slow labor and prolong discomfort, which would at least partially explain the link between psychological distress and traumatic childbirth experiences (Saxbe, 2017).

Birthing parents who report adverse childhood experiences and interpersonal trauma histories are significantly more likely than those without trauma histories to endorse traumatic childbirth experiences (Choi & Sikkema, 2016; Osofsky et al., 2021; Shamblaw et al., 2021; Soet et al., 2003). In fact, one study found that birthing parents’ interpersonal trauma histories predicted childbirth-related PTSD symptoms above and beyond prenatal psychopathology, lack of perceived social support, and labor pain (MacKinnon et al., 2018). This finding underscores the potency of *preconception* trauma histories as predictors of adverse and traumatic childbirth. There is a wealth of literature linking childhood maltreatment experiences – particularly, childhood sexual abuse – with traumatic childbirth, and the mechanisms facilitating these relations warrant further investigation (Lev-Wiesel et al., 2009; Shamblaw et al., 2021). Mechanisms that have been tested empirically include trauma-related schemas pertaining to helplessness and low perceived safety (Ayers, 2004; Iles & Pote, 2015; King et al., 2017; Soet et al., 2003); intimate partner violence during pregnancy (Do et al., 2022; Oliveira et al., 2017); maladaptive physiological stress responsivity during pregnancy (Yehuda & Meaney, 2018); re-traumatization, physical pain, and dissociation during childbirth (Choi & Seng et al., 2016; Ford & Ayers, 2011; Goutaudier et al., 2012; Reed et al., 2017); and gut microbial communities that potentiate atypical inflammatory and glucocorticoid stress responses (Hantsoo et al., 2019). The multidimensionality of these mechanisms highlights the relevance of developmental psychopathology conceptualizations of childbirth.

Finally, birthing parents’ perceptions of being insufficiently cared for or ignored by healthcare staff and inadequately educated or consulted about childbirth procedures are also associated with appraisals of childbirth as traumatic (Ayers, 2004; De Schepper et al., 2016; Soet et al., 2003). One qualitative study found that birthing parents reported feeling coerced to comply with unwanted childbirth procedures in order to satisfy hospital staff’s needs over their own (Reed et al., 2017). Some of these birthing parents described themselves as dehumanized objects of learning for healthcare staff. This latter point has been communicated powerfully by the Black birthing parent community, given that enslaved Black birthing parents often served as “experimental subjects in the development of the [reproductive healthcare] field” (Cooper Owens & Fett, 2019, p. 1342). Indeed, limited access to culturally responsive healthcare has been theorized as a contributor to adverse childbirth experiences (Dunkel Schetter, 2011). For example, among pregnant Latinx adolescents, low levels of acculturation have been associated with more subjectively traumatic childbirth experiences, and this association may result in part from barriers to culturally responsive healthcare (Anderson & Strickland, 2017). Among Black birthing parents, experiences with racism during prenatal care have been associated with preterm birth above and beyond depressive symptoms (Dole et al., 2004). Moreover, a recent large-scale longitudinal study found that risk for preterm birth was especially heightened among Black sexual minority birthing parents, even after accounting for preconception and prenatal risk factors and socioeconomic indicators (Everett et al., 2021). This study found that identifying as bisexual or lesbian was *protective* among White birthing parents, which highlights the importance of considering intersectional identities in reproductive health inequities and discriminatory healthcare practices.

## Multifinal postnatal health trajectories following childbirth

Following childbirth, hormone levels plummet (Schaffir, 2016). Oxytocin and prolactin are two hormones that do not exhibit sudden reductions, as they have been found to play critical roles in facilitating positive mother–infant bonding and inducing milk letdown for breastfeeding. However, birthing parents who endured adverse and traumatic childbirth experiences, such as prolonged labor or emergency Cesarean birth, are more likely to exhibit elevated stress hormones, abnormal oxytocin release patterns, and lower levels of prolactin (Beck & Watson, 2008). Atypical hormone changes and stress responsivity can impede milk letdown and hinder birthing parents’ abilities to breastfeed (Beck & Watson, 2008; Schaffir, 2016). Given that prolactin and oxytocin promote estrogen suppression, which reduces corticotropin-releasing hormone production, birthing parents’ difficulties with breastfeeding may further increase physiological stress responsivity and propel a maladaptive neurobiological cycle.

It has been theorized that birthing parents who are particularly sensitive to atypical hormone changes following childbirth may also be more vulnerable to postpartum psychopathology, such as depression, anxiety, and posttraumatic stress disorder (Osborne et al., 2019; Schiller et al., 2015), which has bidirectional associations with breastfeeding challenges and pain (Iles & Pote, 2015). Breastfeeding-related pain and postnatal psychopathology have been found to elicit feelings of shame and guilt, impede birthing parents’ bonding with their infants, and increase fear of future childbirth (Iles & Pote, 2015; James, 2015). Some birthing parents have reported that they persist through breastfeeding challenges due to strong desires to “prove” themselves as capable mothers and escape from ruminative thoughts and emotional pain (Ayers, 2004; Beck & Watson, 2008). Other birthing parents describe breastfeeding following childbirth trauma as another form of physical violation (Beck & Watson, 2008). Birthing parents with interpersonal trauma histories may be especially vulnerable to flashbacks and dissociation while breastfeeding, which may further challenge their abilities to bond with their infants (Haagen et al., 2015; Iles & Pote, 2015).

Black birthing parents’ endorsements of breastfeeding pain and other physical injuries following traumatic childbirth are significantly more likely to be overlooked or minimized by healthcare staff than are those of White parents (Cooper Owens & Fett, 2019; Greenwood et al., 2020). Black, Latinx, and low-income birthing parents also are significantly less likely to receive breastfeeding education and support in the face of those challenges, which increases their risk for postpartum psychopathology (Anstey et al., 2017; Lara-Cinisomo et al., 2016). These disparities in support following childbirth are concerning because these individuals are significantly less likely than are White, affluent birthing parents to be screened for PPD, despite their increased risk (Rich-Edwards et al., 2006; Sidebottom et al., 2021). Even when birthing parents of marginalized identities are screened and referred for PPD treatment, they often face substantial instrumental barriers to care (Abrams et al., 2009; Davis & Narayan, 2020; Lara-Cinisomo et al., 2016).

### Protective factors and adaptive coping: the role of social support

In the face of adverse and traumatic childbirth experiences, birthing parents can exhibit profound coping and adaptive health trajectories. Research has focused most extensively on the protective effects of social support from family members and partners (Saxbe, 2017). Indeed, social support is “one of the most robust correlates of better maternal mental health” and is associated with lower depressive and anxiety symptoms among birthing parents across racial, ethnic, and income groups (Atzl et al., 2019; Davis & Narayan, 2020, p. 1632). Perceived social support also has been found to influence birthing parents’ health at 3 months postpartum more strongly than negative self-perceptions and traumatic appraisals of the childbirth experience (Ford et al., 2010). Social support reduces cortisol levels and perceptions of pain, which is critical for birthing parents who endured physically traumatic childbirth and/or are experiencing breastfeeding challenges (Heinrichs et al., 2003; Jewell et al., 2015; Moyer et al., 2004). Social support also facilitates opportunities for sleep among birthing parents. Infant care needs and hormone changes substantially disrupt sleep during the first few postpartum months, but consolidated, high quality sleep is possible and vital for lowering cortisol levels, alleviating pain perceptions, and promoting emotion regulation (Bazalakova et al., 2014; Lillis et al., 2018; Owais et al., 2018; Sivertsen et al., 2017). Research indicates that breastfeeding may facilitate more restorative sleep, as well, because of the positive association between prolactin and slow wave sleep (Bazalakova et al., 2014). Thus, there are interactive relations among these protective factors (i.e., social support, breastfeeding, and sleep) that warrant further investigation.

Although invalidating interactions with healthcare staff can increase risk for traumatic childbirth, supportive interactions during and following childbirth can be protective. For instance, providers who facilitate skin-to-skin contact between birthing parents and their infants – no matter how brief – help to promote bonding and breastfeeding and reduce anxiety and depressive symptoms (Bystrova et al., 2009). Comprehensive, compassionate, and personalized infant feeding education from providers also enables these adaptive outcomes (Beck & Watson, 2008; Cricco-Lizza, 2005). Supportive obstetric teams who invite birthing parents and partners to ask questions and participate in decision-making promote perceptions of control during and following childbirth, even in the face of unexpected medical complications. Birthing parents’ perceptions of control have been associated with higher ratings of fulfillment, satisfaction, and emotional well-being following childbirth, which has long-term implications for their and their infants’ health (Ayers, 2004; King et al., 2017). Notably, White and high-income birthing parents often experience more control and decision-making privileges during childbirth (Bakhtari et al., 2019). These healthcare inequities have prompted more research on the benefits of working with doulas and community health workers for reducing birth-related pain, lowering rates of Cesarean births, and rectifying disparities in maternal morbidity and mortality rates (Hodnett et al., 2013; McCloskey et al., 2021; McGrath & Kennell, 2008).

## Implications for research and clinical practice

### Research

**Figure 1.**
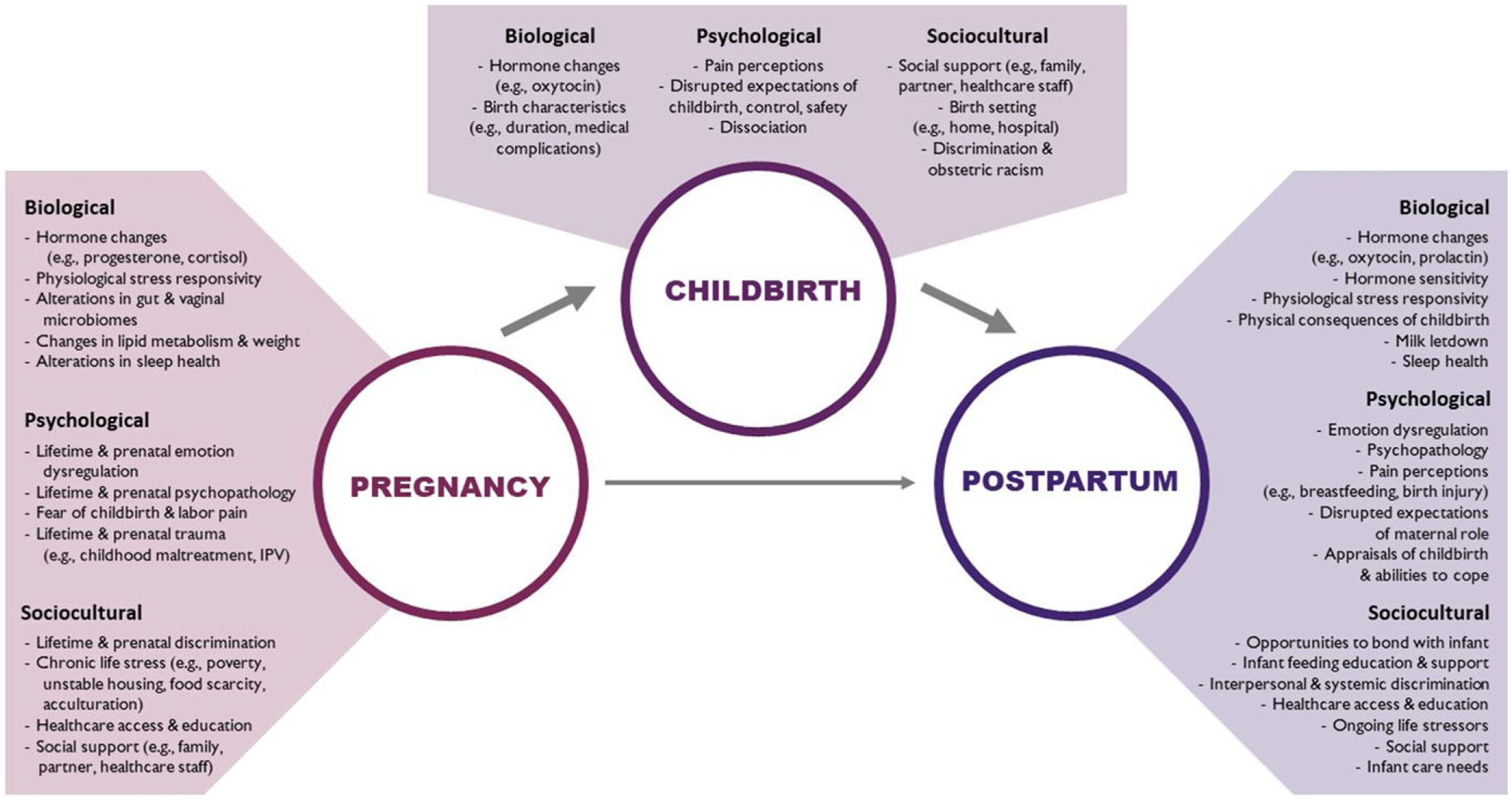
A conceptual model of childbirth as a pathway by which prenatal experiences may indirectly influence postnatal health trajectories. Biological, psychological, and sociocultural factors interact dynamically during each of these developmental stages.

The conceptual model presented in Figure 1 illustrates relations across multiple levels of analysis that can inform testable hypotheses of childbirth as a mediator of the perinatal transition. We hope that this model not only illuminates the importance of including childbirth in perinatal research, but aids scholars in conceptualizing childbirth and the perinatal transition from a truly biopsychosocial perspective versus one that characterizes White, well-educated, and middle-class experiences as “typical” (and all others as “atypical”). Furthermore, we hope that presenting this model within a developmental psychopathology framework underscores the significance of a developmental perspective on childbirth and perinatal health (for a similar developmental perspective on pregnancy-related morbidity and mortality among Black US birthing parents, see Lin & Appleton, 2022).

Our model offers numerous, exciting avenues for future inquiry. For instance, researchers may consider examining how prenatal changes in the vaginal microbiome influence childbirth experiences and subsequent postpartum mood and stress responsivity, as well as how daily experiences with racism moderate those associations. Similarly, researchers may consider investigating the influences of lifetime and healthcare-related discrimination on childbirth experiences and postnatal sleep health. Including childbirth in perinatal research warrants systematic measurement across studies, so more research is needed on valid measurement of the birth experience (e.g., City Birth Trauma Scale [Ayers et al., 2018]; Birth Experiences Questionnaire [Saxbe et al., 2018]; see also Berger et al., 2021). Rigorous measurement is especially important for accurately assessing the prevalence of childbirth-related postnatal psychopathology, such as PTSD (Williams et al., 2022). More research also is needed on multilevel protective factors and adaptive health trajectories following traumatic childbirth (for a systematic review on perinatal protective factors and guidance for future research, see Atzl et al., 2019). Finally, given what psychological science has illuminated about biosocial coregulation among romantic dyads (e.g., Butler & Randall, 2013; Helm et al., 2014; Saxbe & Repetti, 2010), more research is needed on partners’ childbirth experiences (Saxbe, 2017). This area of research may advance our limited understanding of partners’ risk for postnatal psychopathology (Paulson & Bazemore, 2010).

Ideally, research addressing the interacting systems presented in our model will comprise longitudinal data sets that can account for idiosyncratic, nonlinear, and bidirectional relations (Suls & Rothman, 2004; for a recent longitudinal investigation of maternal depressive symptoms following preterm birth, see Roubinov et al., 2022). Indeed, developmental psychopathology evolved from “dissatisfaction with static models of pathology” (Mascolo et al., 2016, pp. 665). Dynamic systems (DS) models offer one promising approach for handling data sets needed for multilevel developmental psychopathology research on childbirth and the perinatal transition. DS theory is a meta-theoretical framework that accounts for complex relations among variables over time (Granic, 2005; Mascolo et al., 2016). DS models are well suited for illustrating how adaptive and maladaptive psychological functioning emerges and changes in response to lower-order biological, psychological, and sociocultural processes (Mascolo et al., 2016). In addition, DS models are ideal for examining transactional relations during developmental periods of heightened neurobiological plasticity because of their attention to rapid and enduring reorganization of states of functioning (Kaliush et al., 2021). Ambulatory assessment (e.g., Lazarides et al., 2021; Mendez et al., 2019; Sanjuan et al., 2019), digital phenotyping (e.g., Vlisides-Henry et al., 2021), and natural language processing (e.g., De Choudhury et al., 2013; Gonzalez-Hernandez et al., 2017) are just a few methodological techniques that could advance dynamic research on childbirth and the perinatal transition.

### Clinical practice

Heightened neurobiological plasticity during the perinatal period may exacerbate birthing parents’ risk for psychopathology following childbirth. However, this developmental sensitivity also presents a window of possibility for promoting resilience and long-term adaptive health (Davis & Narayan, 2020; Kim, 2021; Saxbe et al., 2018). Moreover, the perinatal period is unlike any other life stage in terms of frequent contact with the healthcare system and increased motivation for change (Davis & Narayan, 2020). Developmental psychopathology models are ideal for this time period because they shed light on ways to alleviate the emergence of maladaptation while simultaneously promoting adaptation (Cicchetti, 2016). Thus, in addition to offering numerous research directions, our model implies biopsychosocial avenues for preventative intervention that may foster adaptive postnatal health trajectories. This multilevel developmental psychopathology approach is critical to advancing childbirth and perinatal healthcare beyond its predominant focus on pathologizing birthing parents and neglecting broader systemic factors.

MOMCare (Davis et al., 2018), Perinatal Child-Parent Psychotherapy (P-CPP; Narayan et al., 2016), and the Perinatal Mental Health Promotion Model (Fahey & Shenassa, 2013) are a few examples of promising multilevel preventative interventions during the transition to parenthood. MOMCare is a brief interpersonal psychotherapy designed to treat prenatal depressive symptoms among low-income birthing parents of color (Davis et al., 2018; Davis & Narayan, 2020). This intervention is innovative in that it targets marginalized birthing parents and engages them in conversations about barriers to treatment (Davis & Narayan, 2020). P-CPP is adapted from evidence-based CPP and aims to improve mother–child emotional attunement among traumaexposed birthing parents, specifically (Narayan et al., 2016). The Perinatal Mental Health Promotion Model is not an intervention, per se, but rather a transdiagnostic framework for postpartum health promotion (Fahey & Shenassa, 2013). This model aligns closely with the multilevel nature of our model and asserts that adaptive postpartum health “goes beyond merely the absence of medical complications;” rather, it implies that birthing parents are equipped with individual, social, and community resources to successfully transition into motherhood (Fahey & Shenassa, 2013, p. 613). Given that social support may be one of the strongest protective factors for birthing parents during this life transition (Atzl et al., 2019), interventions that align with this model are critical to preserve relationship quality (e.g., Schulz et al., 2006).

These broader themes of cultural responsivity, community engagement, and client-centered care are vital to alleviating potentially traumatic childbirth and promoting adaptive postnatal health. For example, research has shown that even when birthing parents of color are screened for depressive symptoms, they are less likely than White birthing parents to endorse mental health concerns due to previous invalidating healthcare experiences (e.g., providers diminishing their spiritual and self-care practices; Abrams et al., 2009). One solution for effective perinatal healthcare may include moving beyond training for PPD screening and simultaneously include cultural responsivity training (Clayton et al., 2020; FitzGerald & Hurst, 2017; Moreland-Capuia, 2019). Also, dimensionally screening for additional forms of psychopathology risk besides depression, such as emotion dysregulation (Lin et al., 2019) or posttraumatic stress symptoms (e.g., LeBeau et al., 2014), is critical given that over one-third of birthing parents describe their birth experiences as traumatic but do not necessarily meet full diagnostic criteria for PTSD or depression (Beck et al., 2013; de Graaff et al., 2018; Yildiz et al., 2017). Without consistent, dimensional, comprehensive, and culturally responsive screening and follow-up, these birthing parents will never receive adequate perinatal healthcare.

In addition to increasing cultural responsivity and improving mental health screening, outcomes for birthing parents will be enhanced by a shift toward more comprehensive, communicative, and trauma-informed models of “caring” versus biomedical models of “curing” (Hunter, 2006; Sperlich et al., 2017). This paradigm shift is especially important among birthing parents with trauma histories and ongoing psychopathology who are more likely than those without trauma histories to experience modern-day, Westernized childbirth practices as victimizing, violating, and disenfranchising (Choi & Seng, 2014; Ford & Ayers, 2011). This paradigm shift could empower and engage birthing parents by including more client-centered language (e.g., infants are “borne” by the mother versus “delivered” by the provider; Hunter, 2006); increasing perceptions of safety, autonomy, trustworthiness, transparency, and respect (Choi & Seng, 2014; Seng, 2015; Sperlich et al., 2017; Stanton & Gogoi, 2022); and building collaborative, multidisciplinary healthcare teams (e.g., obstetricians, midwives, doulas, psychologists, and social workers; Ickovics et al., 2019). This latter point about multidisciplinary teams is vital for effectively delegating birthing parents’ healthcare needs and has been presented as one component of a solution for reducing racial disparities in perinatal health (Essien et al., 2019).

## Conclusions

Childbirth may be only a fragment of the perinatal period – let alone the life span – but it is a powerful point of change. Biological, psychological, and sociocultural aspects of childbirth have the potential to alter the health course of the entire family, and yet the birth experience is sorely neglected in developmental psychopathology models of the perinatal transition. To address this limitation, we presented a conceptual model that illustrates childbirth as a bridge between birthing parents’ prenatal and postnatal health. Biopsychosocial processes during pregnancy interact to shape the birth experience, which then propels multifinal postnatal health trajectories. We described this model within a developmental psychopathology framework in order to highlight multilevel, dynamic processes across the life span and emphasize the fluid interplay between adaptive and maladaptive functioning. It is incumbent on perinatal scientists to include childbirth in their investigations of maternal, offspring, and family health. Furthermore, prevention and intervention efforts are critical during this life phase and also must address multiple levels of influence. Research and healthcare advancements should occur simultaneously and inform each other, as translational efforts will more effectively prevent traumatic childbirth experiences and promote health and well-being among birthing parents and their children.

## Footnotes

1 We use the term *birthing parents* to refer to those who are pregnant and give birth to a child who they plan to parent. We use this language to acknowledge gender identity and sexual orientation diversity among pregnant people (for commentaries on affirming language in perinatal healthcare, see Duckett & Ruud, 2019; Hunter, 2006).
